# Unexplained Progressive Visual Field Loss in the Presence of Normal Retinotopic Maps

**DOI:** 10.3389/fpsyg.2018.01722

**Published:** 2018-10-15

**Authors:** Christina Moutsiana, Radwa Soliman, Lee de Wit, Merle James-Galton, Martin I. Sereno, Gordon T. Plant, D. Samuel Schwarzkopf

**Affiliations:** ^1^Psychology, School of Social Sciences, University of Westminster, London, United Kingdom; ^2^Division of Psychology and Language Sciences, University College London, London, United Kingdom; ^3^Radio-Diagnosis, Assiut University Hospitals, Asyut, Egypt; ^4^The Institute of Neurology, University College London, London, United Kingdom; ^5^National Hospital for Neurology and Neurosurgery (NHNN), London, United Kingdom; ^6^Psychology, San Diego State University, San Diego, CA, United States; ^7^Department of Psychological Sciences, Birkbeck University of London, London, United Kingdom; ^8^School of Optometry and Vision Science, University of Auckland, Auckland, New Zealand; ^9^UCL Institute of Cognitive Neuroscience, London, United Kingdom

**Keywords:** anopia, population receptive field, conscious perception, vision, retinotopy

## Abstract

Lesions of primary visual cortex or its primary inputs typically result in retinotopically localized scotomas. Here we present an individual with unexplained visual field loss and deficits in visual perception in the absence of structural damage to the early visual pathway or lesions in visual cortex. The subject, monocular from an early age, underwent repeated perimetry tests over 8 years demonstrating severe anopia of the lower hemifield, and a clockwise progression of the loss through her upper left visual field. Her visual impairment was evident in a number of standardized tests and psychophysics, especially in tasks assessing spatial integration using illusory contours. However, her intellectual ability was intact and her performance in some other tasks assessing color vision or object detection in scenes was normal. We employed functional magnetic resonance imaging (fMRI), electroretinography and visually evoked potentials. Surprisingly, in contrast to the participant’s severe anopia, we found no evidence of abnormal function of her early visual pathways. Specifically, we performed retinotopic mapping using population receptive field (pRF) analysis to map the functional organization of visual cortex in the anopic participant and three control participants on two occasions three and a half years apart. Despite the behavioral visual field loss, her retinotopic maps and pRF parameters in visual areas V1–V3 were qualitatively normal. Further behavioral experiments confirmed that this discrepancy was not trivially explained by the difference between stimuli used for retinotopic mapping and perimetry. Structural T1 scans were normal at both time points, and volumetric analysis of white and gray matter tissue on the segmented T1 volumes did not reveal any abnormalities or deterioration over time. Our findings suggest that normal functional organization of early visual cortex without evident structural damage to the early visual pathway as disclosed by the techniques employed in this study does not necessarily guarantee conscious perception across the visual field.

## Introduction

Retinotopically organized scotomas (total blindness) that are restricted to a homonymous portion of the binocular visual field are typically coupled with cortical lesions in the corresponding part of V1 or the afferent inputs to it (Tong, 2003; Papanikolaou et al., 2014). Models of hierarchical organization and functional specialization of visual information processing credit V1 to be the primary relay for most, but not all (Cowey and Stoerig, 1991; Tong, 2003) visual information to higher level areas, which show functional specificity to more complex inputs (Tong, 2003). Lesions in V1, that contains a topographic high resolution map of the visual field, result in visual loss in the corresponding part of the visual field which can appear complete to standard clinical methodology (Holmes, 1918; see also Papanikolaou et al., 2014); lesions in extra-striate cortex on the other hand, which show functional specificity to complex inputs such as color (V4), motion (MT and MST) or object recognition (inferotemporal cortex; Gross et al., 1969; Zeki, 1974, 1977) are commonly linked to more restricted deficits involving sub-modalities of visual perception. According to the classical hierarchical models, total damage to the striate cortex disrupts information transition to extra striate areas that are responsible for conscious vision (Tong, 2003). On the other hand, interactive models suggest that V1 forms dynamic recurrent circuits with higher level areas via which it contributes directly to conscious perception (Lamme and Roelfsema, 2000; Bullier, 2001).

It has been demonstrated on several occasions, that some patients with V1 damage preserve the ability to accurately (above chance levels) respond to visual inputs presented within their clinically absolute visual field defects, even without conscious awareness of visual stimulation (Pöppel et al., 1973; Weiskrantz et al., 1974) a phenomenon described as “blindsight.” Blindsight is usually demonstrated by forced-choice techniques requiring participants to guess the presence, location, orientation or the movement direction of the stimulus at above chance levels (Weiskrantz et al., 1974; Stoerig, 1997). Data of hemidecorticated patients with little evidence of residual visual discrimination abilities and animal studies demonstrate that the phenomenon of blindsight might require an intact extrastriate cortex (Tong, 2003; Silvanto and Rees, 2011).

**FIGURE 1:**
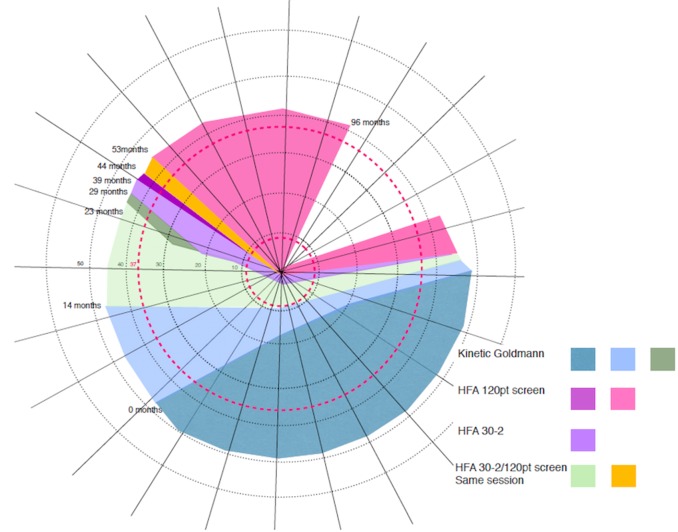
Schematic representation of CW’s progressive visual field loss based on multiple visual field tests^∗^. Over the 8 years period CW showed a constant clockwise progression of visual field loss. Colored areas represent the loss as assessed at the time (in months) of eight visits. At the time of the first visit (0 months) the loss only affected part of the lower visual field (gray color). At the time of the last assessment (96 months after the first visit), the loss progressed to cover most of the visual field, with her vision remaining intact only within a wedge-shaped region in the upper right visual quadrant (blank region in the top right of the figure). Concentric rings around the center of the figure (corresponding to fixation) represent the maximum eccentricity of our two retinotopic mapping experiments (37.5 and 9°).

Here we present a female participant, CW (^∗^CW = clockwise, not her initials) with unexplained vision loss in the absence of retinal or cortical lesions. A variety of perimetric studies tests carried out over the course of 8 years have revealed a relentless clockwise progression of the loss through the upper left visual field crossing the upper vertical meridian, and a moderate anticlockwise progression of the loss in the right hemifield. At the last visit her vision was restricted only within a wedge-shaped region in the upper right visual quadrant (**Figure 1**). In contrast, there is no evidence for structural deficits along the visual pathway, and the functional organization of V1–V3 appears normal. To investigate the source of CW’s vision loss, we employed a couple of psychophysical, behavioral computerized tests and functional neuroimaging. We performed population receptive field (pRF) mapping for retinotopic mapping to assess whether maps in her visual cortex reflect her vision loss in any way.

## Materials and Methods

### CW’s Clinical Profile

At age 8, CW’s left eye was surgically removed because of retinoblastoma, and she wore a prosthesis since that time. At age 42, she became aware of a loss in the lower half of her vision, evident when she was descending stairs. She could not, however, exclude the onset being earlier. A variety of perimetric studies have revealed a relentless clockwise progression of the loss through the upper visual field and a moderate anticlockwise progression of the loss in the right hemifield (see **Figure 1** for a schematic depiction of the progressive loss). At the time of the last visit, CW’s vision remained intact only within a wedge-shaped region in the upper right visual quadrant (**Figure 1**). She is also unable to perceive shapes that require spatial integration such as those defines by illusory contours. Her intellectual ability is normal.

Extensive clinical investigation has indicated that the loss of vision does not result from dysfunction in the early visual pathway. Strikingly, best corrected visual acuity, fundus examination of the retina and the optic disk, electroretinogram using the ISCEV protocol, cortical visually evoked potentials, using the pattern reversal checkerboard and macular and peripapillary retinal nerve fiber layer by spectral domain optical coherence tomography (OCT) were all well within the normal limits. In addition, a structural MRI (see **Supplementary Figure S1**) from the brain and visual pathway revealed no evidence of any macroscopic cortical lesions nor atrophy. Moreover, the gradual clockwise progression of the visual field loss is inconsistent with neglect. Further to her extensive visual field defect findings, CW also exhibits impaired visual processing with regard to spatial integration (**Tables 1** and **2**), being unable to perceive the global shape of simple shapes that presumably rely on spatial integration processes (see **Supplementary Material**: L-POST and Supplementary example stimuli) even though she can reliably describe the orientations of local image components, such as the orientations of the individual grating patches or those of the parallel lines used as a carrier for the illusory contours. Her early and high level visual processing is severely impaired, in particular in relation to spatial integration, as demonstrated by a number of classical visual tests (**Tables 1** and **2**), while her general intellect function is normal (**Table 1**). She was seen on several occasions in the 4 years prior to her last visit, with no significant difference in her performance on these measures and ophthalmological tests performed over this period. Information about the assessments is given below.

**Table 1:** Tests performed in the Ophthalmological Clinic following the standardized protocols.

General Intellectual Function	
NART IQ Equivalent	Average (107)
Language (Graded Naming Test)	Superior (27/30)
WASI Matrices	High average
Verbal Memory (Warrington RMW)	Superior (49/50)
Topographical Memory (Camden Battery)	Average (27/30)
**Early Visual Function**	
Shape Perception	
Efron Test *Shape Discrimination*	Unimpaired
CORVIST *Size Discrimination*	**Impaired (0/2)**
VOSP *Shape Detection*	**Impaired (at chance)**
Orientation (Benton et al., 1978)	**Impaired (13/30 < 1st percentile)**
Color Perception (*Farnsworth 100 Hue Test)*	Average (Error score 83)
**Higher Visual Function**	
Object Perception	
VOSP *Incomplete Letters*	**Impaired (0/20)**
VOSP *Object Decision*	**Impaired (< 1st percentile)**
VOSP *Silhouettes*	**Impaired (1/30 < 1st percentile)**
*Unusual Views* (Warrington and Taylor, 1973)	Unimpaired (17/20)
*Face Perception (Warrington RMF)*	**Impaired (< 5th percentile)**
**Spatial Perception**	
VOSP Dot Counting	Unimpaired (10/10)
VOSP Position Discrimination	**Impaired (12/20 < 1st percentile)**
VOSP Number Location	**Impaired (5/10 < 5th percentile)**
VOSP Cube Analysis	**Impaired (5/10 < 3rd percentile)**
WASI Block Design	Unimpaired (high average)

**Table 2:** Tests performed in the Lab at University College London

Conscious Vision/Blind sight screening	
Stimulus presented in	
intact quadrant (upper right)	Unimpaired
impaired quadrant (lower left)	**Extremely impaired^∗^**
**Early Visual processing**	
Motion Coherence Test	**Extremely impaired^∗^**
Grouping of oriented gratings (Kanizsa)	**Extremely impaired^∗^**
**Midelevel Vision (L-POST) ^∗^ (2013)** Impaired (< 3rd percentile)	
Fine Shape discrimination	**Impaired**
Shape ratio discrimination (Efron)	Unimpaired
Dot Lattices	Impaired
RFF fragment Outline	Impaired
RFF Contour Integration	Impaired
RFF Texture Surfaces	Impaired
Global Motion Detection	**Extremely Impaired**
Kinetic Object Segmentation	**Extremely Impaired**
Biological Motion	**Extremely Impaired**
Dot Counting	**Extremely Impaired**
Figure Ground Segmentation	Unimpaired
Embedded Figure Detection Segmentation	Unimpaired
Recognition Of Missing Part	Unimpaired
Recognition Of Object In Isolation	**Impaired**
Recognition Of Object In Scene	**Impaired**

### Clinical Assessments During the 4 Year Period Conducted in the Ophthalmological Clinic

#### Ophthalmological Assessment

CW’s ophthalmological examination during all visits between 2009 and 2013 conducted in the Ophthalmological Clinic in *Moorfields Eye Hospital* and/or in *The National Hospital for Neurology and Neurosurgery* (NHNN) using the International Society for Clinical Electrophysiology of Vision (ISCEV) protocols and consisted of the following examinations:

• Best corrected visual acuity (BCVA)
• Slit lamp examination
• Fundus examination including optic disk
• Pupil light reflex
• Electroretinogram (2005 ISCEV protocol)
• Visually evoked potentials to high contrast pattern reversal
• Optical coherence tomography of the retina was carried out using the Stratus Zeiss equipment (2008 protocol)
• (Clinical) Magnetic Resonance imaging of the anterior visual pathway

#### General Intellectual Function Tests

These included the following standardized tests: The NART IQ (Nelson and Willison, 1991), the Graded naming test assessing impaired language functioning (McKenna and Warrington, 1983), the Wechsler Abbreviated Scale of Intelligence (WASI) (Wechsler, 1999) test for general cognitive ability, the Warrington Recognition Memory for Words (RMW) (Warrington, 1984) and the Topographical memory using the Camden Battery (Warrington, 1996).

#### Visual Processing via Standardized Tests Commonly Used in Clinical Population

**Early Visual Processing** assessments included tests assessing *Shape Perception* using the Shape (Efron, 1982) and Size Discrimination (James et al., 2001) and the Shape Detection (Warrington and James, 1991) tasks, *Orientation* (Benton, 1962), *Grouping* (Kanizsa, 1979) *and Color Perception* (Farnsworth, 1957). **Higher Visual Functioning** was assessed on *Object* and *Face Perception tests using the* Visual Object and Space Perception battery (VOSP) (Warrington and James, 1991). **Spatial Perception** tests included the Position Discrimination, Number Location, Cube Analysis tests and the Block Design tests (Benton et al., 1978) and the WASI Block design tests (Wechsler, 1999).

### Laboratory Assessments During the Last Two Visits (at 44 and 53 months) at the University College London Lab

In addition to the ophthalmological tests, we tested CW’s performance in the Laboratory on a number of behavioral and psychophysics paradigms. All these tests were performed on a computer system in a darkened lab cubicle, with only CW and the experimenter present. In those tests where CW was asked to make verbal responses data were recorded by the experimenter using a computer keyboard. Throughout the experiments the position of the participant’s head was stabilized using a chin rest at 43 cm distance from the center 288 of a LCD monitor (Samsung SyncMaster 2233RZ). However, unless otherwise specified CW was encouraged to free view the stimuli as best as she could.

#### Computerized Visual Field Test

For the visual field test, the stimulus display was divided into four concentric rings around a central fixation dot (0.23°) in the middle of the screen. The radius of the outside circle subtended 37.5° and all four rings had equal distance between them (radius in steps of 9.375°). The display was also divided into eight polar angles in steps of 45°. The visual stimulus was a ring segment filled with the same ripple stimulus used during retinotopic mapping. It could appear in any of these 32 possible positions. There were two repetitions of each position, with the patterned stimulus present or not, hence there were 64 trials in total, all presented in a pseudo random order. CW was asked to fixate a point in the middle of the screen throughout the experiment and respond positive or negative whether she saw any stimulus present at all the positions tested. False positive and false negative trials were used to evaluate the extent of her visual field. There were two repetitions of this test, one with a long stimulus duration of 9 s and one with a short duration of 500 ms.

#### Blind Sight Screening Test

Some patients with damage in the early visual areas preserve the ability to accurately (above chance levels) respond in a forced choice task to visual inputs in the absence of visual awareness (Stoerig and Cowey, 1997; Cowey et al., 2008). To evaluate whether CW shows evidence of such blindsight, we performed a blindsight screening test. Specifically using a forced choice method, she was asked to discriminate the orientation (vertical or horizontal) of a Gabor, which was randomly presented either in her intact visual field quadrant or in the polar opposite location.

The stimulus was a grating patch (sinusoidal grating convolved with a Gaussian: 18.75° eccentricity, standard deviation 3.77°, wavelength 3.77°). The patch appeared in either the upper right (in different runs at a polar angle of 45° and 30° relative from 3 o’clock position, respectively) or in the lower left quadrant (polar angle of 225° and 210°, respectively). CW was asked to fixate at a point in the middle of the screen throughout the experiment and to discriminate the orientation of the Gabor (vertical or horizontal). Her accuracy at this task was used to evaluate her potential ability to discriminate a stimulus in her scotoma under a forced choice task. We tested CW on three separate runs and there were 200 trials per run in this experiment.

#### Midlevel Visual Function Standardized Tests

During the last visit, CW’s deficit in mid-level vision and perceptual organization was also assessed using the Leuven Perceptual Organization Screening Test (L-POST) (Torfs et al., 2014; Vancleef et al., 2015). The L-POST is a standardized neuropsychological screening test, specifically designed for clinicians and neurophysiologists and freely available online^1^. Specifically, the screening test contains 15 subtests (see Supplementary example stimuli), which cover most of the key processes of mid-level vision, including figure-ground segmentation, local and global processing, shape perception and the ability to use a range of grouping cues including common fate, co-linearity, proximity and closure (Torfs et al., 2014; Vancleef et al., 2015). The test is entirely computerized, and the scoring is done automatically.

#### Visuo-Spatial Integration

To characterize her deficits with spatial integration we aimed to conduct tests using stimuli such as those depicted in Supplementary example stimuli. However, no formal experimental paradigms could be carried out because she was unable to perform the tasks. For instance, while she could reliably discriminate luminance defined shapes (triangles, circles, and squares) she reported not seeing any shapes for stimuli defined by illusory contours formed by abutting lines. Similarly, she would report seeing a circle if the individual grating patches touched but reported only seeing an incoherent jumble of patches as soon as there was any gap between them.

We tested CW’s visuo-spatial integration on the Ebbinghaus illusion experiment in a darkened room. As before, stimuli were presented on a Samsung SyncMaster 2233RZ at a nominal distance of 57 cm but under free viewing conditions. We used two interleaved one-down, one-up staircases to measure the strength of the illusion. We subsequently determined the threshold performance by removing the signal of all reversals outside +/− 2 median absolute deviations from the median across reversals as done previously (Schwarzkopf et al., 2014).

The Ebbinghaus illusion was assessed using a classical illusion stimulus comprising one target disk on a black background (0.6 cd/m^2^) surrounded by an annulus of 16 smaller disks (disk diameter, 0.4°; distance of disk centers from target center, 1.6°), and one target disk surrounded by an annulus of six larger disks (diameter, 3.5°; distance, 3.7°). These two components of the illusion stimulus were presented in left and right halves of the screen on the horizontal meridian centered at an eccentricity of 4.97°. The side on which each component appeared was counterbalanced and pseudorandomized across trials. The target surrounded by small disks was the test stimulus that varied in size, whereas the other target always remained fixed as the reference (diameter, 1.77°). There were two randomly interleaved staircases, one that started with the test being larger (diameter, 2.64°) than the reference, and the other started with the test being smaller (diameter, 1.19°). The stimulus duration was 1 s and CW was instructed to respond which of the targets was larger. Each time she responded correctly/incorrectly, the size ratio of test and reference would decrease/increase (by 0.04 in natural logarithmic units up to the fifth reversal and by 0.02 units afterward).

In addition to the classic Ebbinghaus test described above CW was also tested in 40 trials in another test in which the stimulus duration was 10 s and the test stimuli were always the same size as the reference. If CW showed evidence of contextual modulation elicited by the basic Ebbinghaus illusion, she should always pick the stimulus with the small inducers as the one that appears larger.

#### Psychophysical Tests of Visual Processing

During both lab visits at 44 and 53 months, we also conducted a number of psychophysical experiments in a darkened lab cubicle. These used stimuli generated in MATLAB that were either presented in Psychtoolbox or simply as images in Windows Explorer. Stimuli were presented on a Samsung SyncMaster 2233RZ at a nominal distance of 57 cm but under free viewing conditions so that the viewing distance was chosen by CW for the best possible view of the stimuli.

We wanted to use a standard 2-down, 1-up staircase procedure to estimate her motion coherence thresholds. In each trial, a field of light gray random dots was presented in the center of a black screen. A proportion of dots were signal dots and either moved horizontally to the left or right. The remaining dots were moving in random directions. If dots moved outside of a circular area around fixation (diameter equal to height of the screen), they were regenerated on the other side. Dot lifetime was otherwise unlimited. Dot number (100 or 5), size (diameters approximately 0.2 or 1° visual angle, but again note that viewing distance was not fixed), and speed (0.5–2°/s) were adjusted to various levels on multiple attempts but the result was always the same. Stimulus duration was also adjusted from 500 ms to unlimited. Theoretically, whenever the participant would correctly identify the coherent motion direction in two successive trials, the coherence level (proportion of signal dots) would be reduced by 5% (or 20% when only five dots were used). Whenever they made a mistake, the coherence level would be increased by 5% (20%).

### MRI Methods

Scanning was performed during the last two visits (at 53 and 96 months) and on the same day with the lab assessments at University College London.

#### fMRI Participants

CW (age 48 years at the time of the first fMRI session, left handed), and five healthy control participants (right handed, mean age 43.5 at the time of the visit, two females) with normal or corrected-to normal vision participated in the fMRI part of the study. CW and two of the controls were scanned twice, three and a half years apart. One control participated only in the first fMRI session, and two controls only in the second session. During the first fMRI session, the left eye in all control participants (*N* = 3) was covered throughout the scan with an opaque piece of foam secured tightly with medical tape to match viewing conditions to her monocular vision. A layer of soft cotton wool between the foam and the eye was used to minimize discomfort. Participants gave their informed consent in accordance with the standard procedure of the Birkbeck-UCL Centre for Neuroimaging (BUCNI). Written informed consent was obtained from all participants and all procedures were approved by the University College London Research Ethics Committee.

#### Stimulus Presentation and Task in fMRI Experiments

Participants lay supine inside the bore and viewed stimuli presented on a screen in two different configurations. In the first fMRI session, stimuli were projected onto a front-projection wide screen located inside the bore in front of the participant’s eyes (but viewed only with the right eye as discussed above). The back of the head coil was tilted forward by means of a wooden block placed under its back. This configuration allowed us to stimulate a field of view with a radius of 37.5° visual angle. In the second visit, stimuli were projected on a screen at the back of the bore. In this configuration, the radius of the stimulated field of view was 9° visual angle and participants viewed the screen binocularly through a mirror mounted above the head coil. The fMRI experiments comprised two functional runs for retinotopic mapping, and one run for estimation of each participant’s haemodynamic response function (HRF).

All stimuli were generated in MATLAB R2012a (MathWorks) and displayed using the Psychtoolbox package, 3.0.10, (Brainard, 1997; Pelli, 1997). Stimuli contained a broadband, dynamic, high-contrast “ripple” pattern (see Schwarzkopf et al., 2014) to maximize visual responses. Each mapping run included both rotating wedges to estimate the polar angle as well as expanding and contracting rings to (Brainard, 1997; Pelli, 1997) estimate the eccentricity of pRFs in visual cortex (Sereno et al., 1995; Wade et al., 2002; Wandell et al., 2007).

During the mapping runs, participants fixated centrally. In both visits the wedge stimulus subtended a polar angle of 36°, and, on each functional MRI image acquired, rotated by 18° around the central fixation dot either in clockwise and anticlockwise directions (alternating with scanning runs). A full cycle was achieved within 60 s with a total of cycles per run (three cycles only in second visit). The rings were expanding or contracting alternating with scanning runs. Their radius was changed on a logarithmic scale in 16 (12) steps, and there were 10 (5) repetitions per run. In the end of each mapping run a blank screen was presented for 20 (15) volumes. Each run was preceded by three additional volumes during which a blank screen was presented. Thus total run duration was 549 s (234 s), corresponding to 78 scanning volumes in total per run. Overall there were two runs per visit, one for clockwise rotation/expanding rings and one for anticlockwise rotation/contracting rings. During the HRF estimation run, we presented the full-scale version of the ripple pattern, i.e., a circular region with a radius of 37.5° (9°) visual angle around fixation. In each trial, the stimulus appeared for one volume (3 s) followed by a blank period of 27 s. There were 10 trials in the run. The fixation dot in all runs was a blue circle with diameter: 1° (0.23°). In addition, there was an annular region with 2.4° (0.55°) diameter within which the contrast of mapping stimulus was ramped up linearly. Throughout the run a low-contrast “radar screen” pattern covered the entire screen to facilitate fixation stability. After every 200 ms the fixation dot could change color to purple with a probability of 0.05 and then change back to blue after 200 ms. Color changes would never occur in immediate succession. To ensure constant fixation and to maintain attention participants were instructed to press a button on an MRI-compatible response box whenever they noticed a color change.

#### MRI Data Acquisition

In both MRI sessions image acquisition was performed at the BUCNI Siemens Avanto 1.5-T MRI scanner using a 32-channel head coil. Because the top half of the coil restricted the participants’ field of view, it was removed during the functional scans leaving 20 effective channels covering middle and posterior part of the head. For the functional data we used a 2.3 mm isotropic echo-planar imaging sequence that was designed to optimize signal detection and reduce dropout in the visual cortex. Each volume comprised of 30 transverse slices oriented parallel to the calcarine sulcus, with no interslice gap, acquired in an interleaved sequence (TR = 3 s, TE = 42 ms; field of view = 221 mm; matrix size = 96^∗^96). To correct for the inhomogeneity of the static magnetic field we acquired B0 fieldmaps to be used in the unwarping stage of data preprocessing. In addition, we acquired a fast T1-weighted MP-RAGE scan to facilitate coregistration of the functional and anatomical images. We also acquired a high resolution MP-RAGE three-dimensional T1-weighted structural scan (176 slices, TR = 2730 ms, and TE = 3.57 ms) of the whole brain, this time using the complete 32-channel head coil.

#### MRI Data Analysis

##### Pre-processing

The first three volumes (9 s) of each functional run were discarded from any further analysis to allow the signal to reach equilibrium. Preprocessing of the remaining images involved intensity bias correction, realignment and unwarping using a B0 fieldmap in SPM8 (Wellcome Trust Center for Neuroimaging^2^,). We first coregistered functional images to the fast T1 structural scans acquired without the front part of the head coil, and then all data was registered to the T1 structural scan acquired with the whole 32 channel coil.

##### Cortical segmentation/reconstruction

This second T1 structural scan was used for segmentation and cortical reconstruction. A 3-D model of each cortical hemisphere (right and left) of each subject was created using an automated procedure implemented in FreeSurfer software v.5.3.0^3^. First, segmentation was performed in order to separate white and gray matter as well as the pial surface and then each hemisphere was inflated. To ensure good extrastriate areas coverage our region of interest included the occipital pole but also parts of the parietal and temporal lobe up to posterior central gyrus. Next, we projected all the fMRI data onto the cortical surface model. We calculated the middle position between each vertex comprising the gray-white matter (WM) surface and the corresponding vertex in the pial surface. Then we transformed these coordinates back into the voxel coordinates in the functional data and extracted the time series for each vertex. For each vertex and separately for each run we then z-standardized the time series by subtracting each time series from its mean and dividing by its standard deviation and applied linear detrending to correct for slow signal fluctuations.

##### Hemodynamic response function

The HRF in each participant was estimated using the same method described previously (Schwarzkopf et al., 2014). In brief, the time series was averaged across each of the 10 repetitions in the HRF run. Because the data were now divided by hemisphere this was done separately for each cortical hemisphere. Only vertices that showed a mean response minus standard error greater than zero during the first half of the trial were included in this analysis while other vertices were discarded as visually unresponsive. A two-gamma function with amplitude, peak latency, undershoot latency, and peak/undershoot amplitude ratio as free parameters was fitted to the average HRF data, and this function was subsequently used for the pRF analysis.

##### pRF model based analysis

The time series from the two mapping runs were concatenated. pRF parameters were estimated using our freely available MATLAB toolbox for pRF mapping analysis^4^, using a forward modeling approach similar to that described by Dumoulin and Wandell (Dumoulin and Wandell, 2008). Specifically, we employed a two-dimensional Gaussian described by four parameters: two encoding the space: x, y (pRF center in Cartesian coordinates relative to fixation), σ (the standard deviation of the Gaussian) denoting the spread of the receptive field in visual space) and β (the response amplitude) (see also (de Haas et al., 2014; Schwarzkopf et al., 2014; Alvarez et al., 2015; Moutsiana et al., 2016). The analysis proceeded in the following steps: First, the model was created on the prior knowledge of the stimulus aperture presented for each stimulus configuration, and the assumption of a simple Gaussian receptive field followed by a three dimensional search space of possible combinations of location and receptive field size. The maximal eccentricity of pRFs in the search space was 1.5 times that of the outer stimulus eccentricity. This search space was then sampled for candidate locations in X and Y and σ values. We estimated these parameters for the time series at each vertex of the sampled cortical surface, restricted to an occipital region of interest delineated manually on the inflated cortical surface. For each vertex and separately for each run we applied linear detrending and then standardized the time series by subtracting each time series from its mean and dividing by its standard deviation. This differs but it is comparable to the procedure used by Dumoulin and Wandell (Dumoulin and Wandell, 2008) in which time series are normalized as percent signal change, with high intersession reliability for both eccentricity and polar angle estimates (van Dijk et al., 2016).

The fMRI time series was predicted as the overlap between the pRF model and the sequence of visual field locations stimulated during each scanning volume and convolving this prediction with the participant’s hemodynamic response function measured in the separate HRF run (see above). The optimal pRF parameters were estimated using a coarse-to-fine procedure in which a pRF model is first fitted to heavily smoothed data using an extensive grid search by maximizing the correlation between prediction and data (omitting the β parameter), and then using the fitted parameters for an optimization procedure minimizing the residuals between prediction and unsmoothed data using all four pRF parameters (de Haas et al., 2014; Schwarzkopf et al., 2014; Alvarez et al., 2015; Moutsiana et al., 2016).

##### Control pRF analysis

In addition to the full stimulus pRF method, we also analyzed data using a stimulus model in which locations roughly corresponding to the CW’s scotoma (all visual quadrants except for the upper right) were masked out. This analysis was performed for both CW and one of the controls.

##### Delineation of retinotopic maps

Visual regions were delineated manually in Freesurfer by displaying pseudo-color coded maps of polar angle and eccentricity maps calculated from the pRF locations. We delineated V1–V3 according to standard criteria using the reversals in the polar angle map (DeYoe et al., 1994; Sereno et al., 1995; Engel et al., 1997).

##### Volumetric calculation of gray and white matter

To acquire volumetric measures of gray and WM tissues we used the default settings of the DARTEL approach (Ashburner, 2007) using the Statistical Parametric Mapping software^5^ (SPM8) implemented in Matlab R2015b (MathWorks). Preprocessing included segmentation of the images into WM, gray matter (GM), and cerebrospinal fluid (CSF) using the standard unified segmentation option implemented in SPM8. To allow comparisons between CW and controls we also applied the DARTEL approach for registration, normalization, modulation and smoothing (8 mm full width half maximum Gaussian kernel). Each voxel in the resulting images represents an absolute amount of brain volume, equivalent to the brain volume per unit prior to normalization. For the CW data only we also carried out pairwise registration and segmentation using the longitudinal data steps for preprocessing (SPM12). In addition, we calculated the gray and WM volume for all segmented tissues using a script^6^, which returns image totals (sum over all voxels), in mmˆ3 (=0.001 ml).

## Results

### Clinical Assessments During the 4 Year Period Conducted in the Ophthalmological Clinic

#### Ophthalmological Assessment

CW’s scotopic rod specific ERGs attained amplitudes of approximately 125 μV. Maximal ERG a- and b- wave amplitudes were 250 and 350 μV, respectively. Oscillatory potentials were present. Averaged photopic 30 Hz flicker ERGs had implicit times and amplitudes of 24 ms and 95 μV, respectively. Transient photopic ERG a- and b-wave amplitudes were 35 and 150 μV. Long duration ON OFF ERGs and S-cone specific ERGs were unremarkable.

At the time of testing multifocal ERG was not available. However, it is very unlikely that such extensive visual field loss could occur without affecting the Ganzfeld ERG – the above results are all well within normal limits apart from the maximal ERG is low normal. Visually evoked potentials to high contrast pattern reversal revealed a major positive component with an amplitude of 8 μV at 114 msec, which is within the normative range (the laboratory’s 95% confidence limits for the P100 VEP latency are 105 to 115 msec. Note this can vary depending on protocols and equipment (Marmor et al., 2009; Odom et al., 2010). Hemifield VEPs were not carried out but there was no hemisphere asymmetry as would be expected from the visual field loss if due to retina or anterior visual pathway dysfunction.

Optical coherence tomography revealed normal retinal morphology. Retinal nerve fiber layer thickness on the peripapillary scan was well within normal limits in all sectors. It would be extremely unlikely that an anterior visual pathway disorder could occur without resulting in some thinning of the nerve fiber layer.

Magnetic Resonance imaging of the anterior visual pathway revealed no abnormality other than what would be expected from the enucleation of the left eye, the right optic nerve has normal thickness and signal properties throughout. The chiasm and optic tracts are thin compatible with loss of 50% of the total number of nerve fibers from total loss of the left optic nerve.

#### General Intellectual Function Tests

In all the tests CW intellectual performance were within the average levels or high average levels as summarized in **Table 1**.

#### Visual Processing Standardized Tests

However, her visual processing found to be seriously impaired. Specifically, in most tests assessing shape perception, object recognition, face perception and spatial perception she scored below the 5th percentile (see details in **Table 1**).

### Laboratory Assessments at University College London

#### Computerised Visual Field Test

We then confirmed the schematic map (**Figure 1**) of the clinical perimetry results using a computerized visual field screening. CW was unable to detect any stimuli presented outside of her intact visual field quadrant even when using the same high contrast stimuli used for retinotopic mapping. Using these stimuli, she correctly detected the presence/absence of stimuli inside the upper right visual quadrant but always responded “absent” if stimuli were shown outside of this region. This was the case both for short (500 ms) and long (9 s) stimulus presentations.

#### Blindsight Test

Her accuracy at this task was almost perfect (100, 83, and 95% correct, respectively, in three separate sessions) when the Gabor was presented in her intact quadrant but near chance level (63, 57, and 43%) when presented in the polar opposite location. We used a binomial Bayesian hypothesis test^7^ to assess if this performance was different from chance levels. The Bayes factors (BF01) for the null (i.e., guessing) over the alternative (blindsight) hypothesis in the three runs were 1.5, 3.7, and 3.3, respectively. While this constitutes only weak evidence for the null hypothesis, it suggests that CW did not exhibit any blindsight in the defective part of her visual field.

#### Midlevel Visual Function Tests- L-POST Screening

We compared CW’s data to the data of different aged matched healthy control sample (*N* = 1376) see (Torfs et al., 2014) for methodological details). She scored 33 out of 75 correct, thus 44.01%. Importantly, in thirteen out of the fifteen subtests CW scored below the threshold of the 10th percentile, which indicates severe deficit in perceptual grouping and integration. See Supplementary L-POST for detailed performance on each subtest.

#### Ebbinghaus Illusion

CW’s Ebbinghaus illusion strength was found to be only 0.02. This was significantly smaller [*t*(11) = 7.45, *p* = 0.00001] than the perceived illusion data of 12 healthy normal controls (five females, mean = 0.195, SEM = 0.024) who participated in the same experiment for the purpose of a different study (Schwarzkopf et al., 2014). In addition, in the test where the test stimuli were fixed, CW performed at chance, which suggests no illusion was present.

#### Psychophysical Tests of Visual Processing

Only a small number of these experiments were completed because CW was unable to perform the tasks. She performed accurately and reliably whenever motion coherence was 100% but as soon as coherence dropped below this she started guessing and reported subjectively perceiving only random or jumbled motion. Thus, her discrimination thresholds in all tests were at or just below 100% coherence. A normal participant should be capable of performing such a task simply by tracking a number of dots. CW’s inability to perform that task rules out any strategy like this. Even when presented with only five large dots she was unable to correctly determine the direction if four dots moved coherently in one direction and the remaining dot moved randomly.

### MRI Data

Careful examination of the high resolution (1 mm) structural scan (see **Supplementary Figure S1** for the most recent T1 scan) revealed no visible brain abnormalities.

**FIGURE 2:**
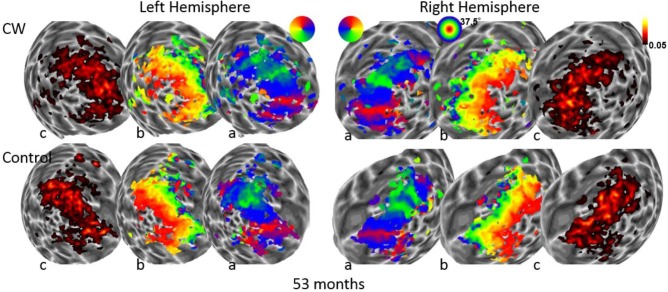
Maps for polar angle **(a)**, eccentricity **(b)**, and R2 of model fits **(c)** on a spherical model of the gray-white matter boundary of the occipital lobe. Maps for CW and one of the control participants are shown from the first retinotopic mapping session at 53 months using a wide field stimulus (37.5° maximal eccentricity). All maps are thresholded at 0.05.

**FIGURE 3:**
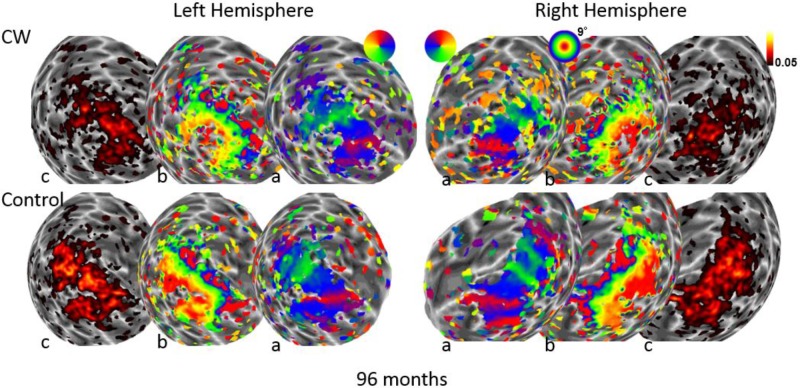
Maps for polar angle **(a)**, eccentricity **(b)**, and R2 of model fits **(c)** on a spherical model of the gray-white matter boundary of the occipital lobe. Maps for CW and one of the control participants are shown from the second retinotopic mapping session at 96 months using a narrow field stimulus (9° maximal eccentricity). All maps are thresholded at 0.05.

**FIGURE 4:**
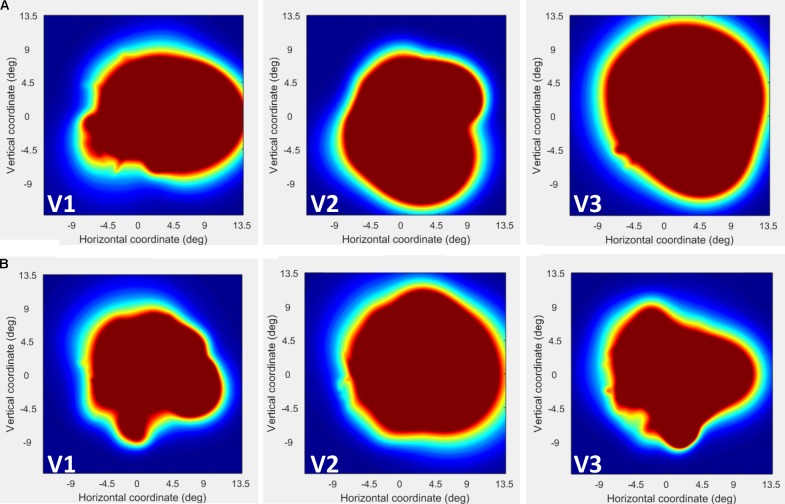
Visual field (VF) coverage plots plotted separately in V1–V3 for CW **(A)** and the control participant **(B)**. This plots the sum of all pRFs in each area (V1–V3) as an estimate of the VF coverage. As seen the coverage for CW is constant across the whole visual field.

We next used the pRF mapping method (Schwarzkopf et al., 2014; Moutsiana et al., 2016) to perform retinotopic mapping and to further study the functional organization of the early retinotopic visual areas. For each voxel, we estimated the pRF, the range of the visual field locations that can drive its response (see Methods). We compared macroscopic maps and the spatial selectivity in the visual cortex of CW and three healthy controls. Scanning was performed during the last two visits (at 53 and 96 months, see **Figure 1**). We analyzed visual areas V1–V3 in both hemispheres. Because the only preserved part of CW’s visual field is in the upper right visual quadrant, it was critical to compare the responses in dorsal and ventral areas in the left hemisphere, which represent the lower and upper right visual field quadrants, respectively – that is, the last remaining intact visual field and the quadrant where vision has been lost the longest. If CW’s visual field loss were related to visual cortical dysfunction, she should show abnormal responses in left dorsal regions corresponding to the lower right quadrant. Strikingly, however, CW’s retinotopic maps showed general normal appearance with no area of abnormal retinotopic organization or areas of absent response in either the polar angle (**Figures 2a**, **3a**) or the eccentricity maps (**Figures 2b**, **3b**) and no differences in the model goodness of fit for these regions (**Figures 2c**, **3c**). Furthermore, the visual field coverage in V1–V3 for CW is constant across the whole visual field (**Figure 4**).

**FIGURE 5:**
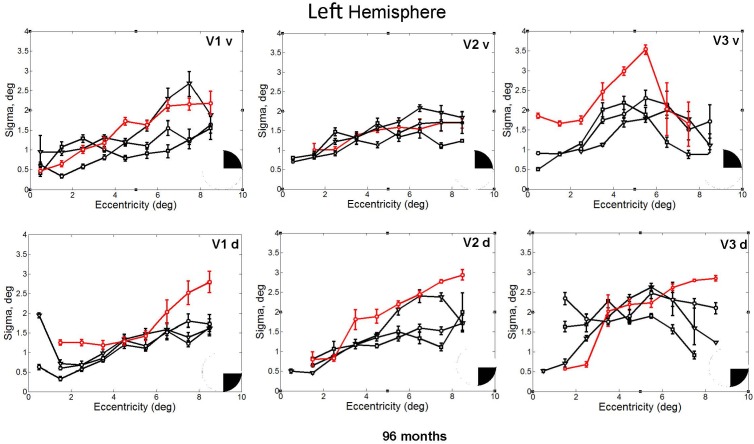
pRF size (σ) plotted against eccentricity for the ventral and dorsal early visual areas. Red color corresponds to CW, black color, to the three controls. Symbols denote the mean in each eccentricity band. Error bars denote 1 standard error of the mean. The black triangle in the circle represents the visual field quadrant that each visual area corresponds to the most recent perimetry tests (at 96 months, see also **Figure 1**) demonstrate that CW’s vision remained intact only within a wedge-shaped region in the upper right visual quadrant (thus corresponding to ventral visual areas).

**FIGURE 6:**
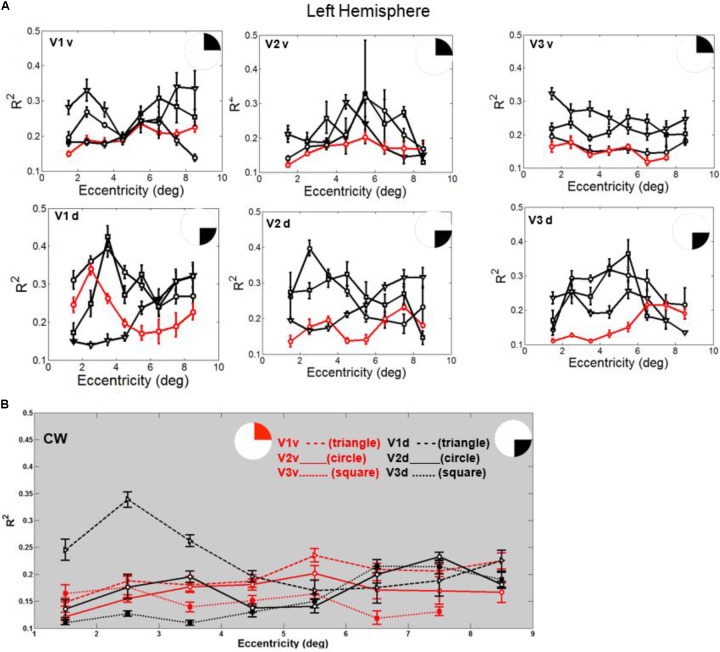
**(A)** Goodness of fit (R^2^) plotted against eccentricity for the ventral and dorsal early visual areas. Red color corresponds to CW, black color, to the three controls. Symbols denote the mean in each eccentricity band. Error bars denote 1 standard error of the mean. **(B)** CW’s data: Goodness of fit (R^2^) plotted against eccentricity for the ventral (red) and dorsal (black) early visual areas.

**FIGURE 7:**
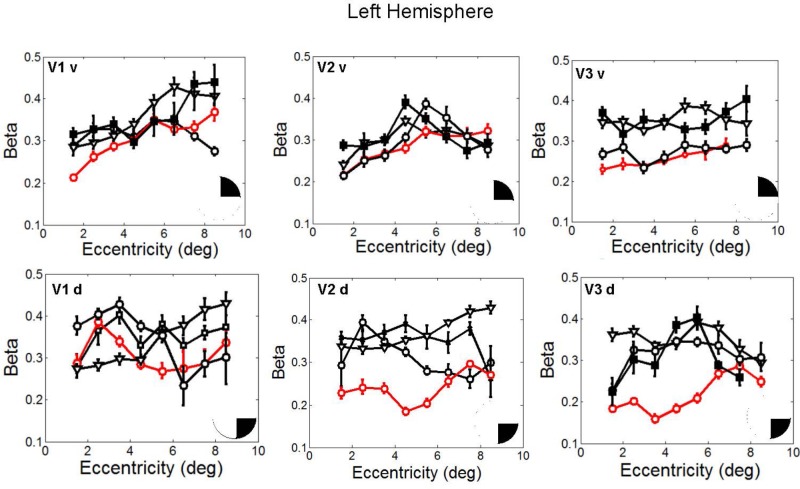
Response amplitude (β) estimates plotted against eccentricity for the ventral and dorsal early visual areas. Red color corresponds to CW, black color, to the three controls. Symbols denote the mean in each eccentricity band. Error bars denote 1 standard error of the mean B. Response amplitude estimates for CW’s ventral and dorsal visual areas. Responses from the dorsal areas correspond to the blind visual field.

**FIGURE 8:**
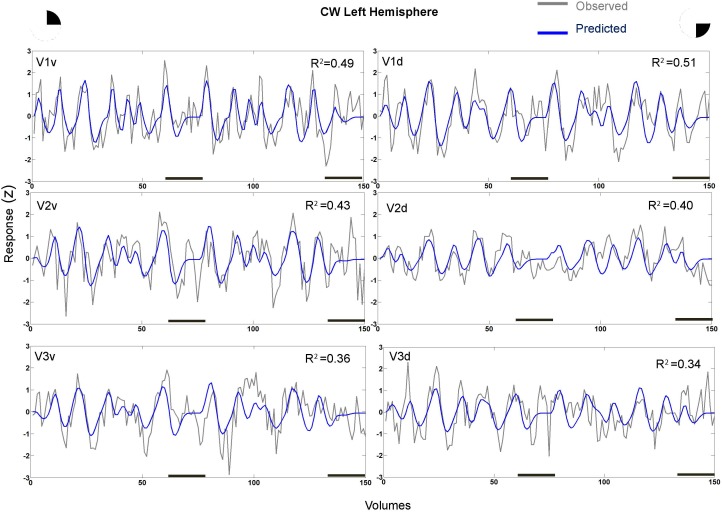
Sample time-series and best-fitting model prediction for example 711 vertices (cortical surface element) in ventral and dorsal V1–V3 areas from CW’s left hemisphere. The dorsal areas correspond to the blind visual field.

In fact, the spatial spread (sigma, **Figure 5**), the goodness of fit (**Figure 6**) and the response amplitude estimates (beta, **Figure 7** and **Supplementary Figure S2**) of pRFs measured in the second retinotopic mapping experiment and plotted against eccentricity in ventral and dorsal parts of left V1–V3 showed that CW’s data were generally comparable to the data of the three control participants. Specifically, for most of the dorsal (corresponding to the most affected part of the visual field) regions CW’s data overlapped with the data of the three controls. Only in CW’s left dorsal V2 and V3 and in right ventral V2 was the response amplitude considerably below the level of controls. Note, however, that pRF parameter estimates can vary substantially between participants (Dumoulin and Wandell, 2008; Binda et al., 2013) even in the normal, healthy population and normal map retinotopic architecture was clearly preserved even in those brain regions. Sample time-series from example vertices in dorsal and ventral early visual areas that correspond to either her blind and preserved visual fields further showed that CW’s responses to visual stimulation generally matched the ones predicted by the pRF model (**Figure 8**).

pRF that do not incorporate the extent of a participant’s scotoma may result in artifactual model fits and may thus bias the pRF parameters and thus the maps (Binda et al., 2013). While it is difficult to conceive how such an explanation can produce *qualitatively normal* maps in the presence of such an extensive visual field loss in this case, we nonetheless performed a control analysis. When we fitted a pRF model based on stimuli in which the portion of the visual field corresponding CW’s scotoma (that is, all but the upper right visual quadrant) was masked out, maps in all the visual areas for both her and a control participant were unsurprisingly severely distorted, possibly with the exception of the ventral left visual cortex where the stimulus remained (**Supplementary Figure S3**). In particular, pRFs in the right hemisphere (corresponding to the left visual field) were mostly estimated to be in the ipsilateral hemifield. These results rule out the (unlikely) explanation that the normal appearance of her retinotopic maps was due to a pRF model fitting artifact in the presence of a wide-spread scotoma.

#### Volumetric Calculation of Gray and White Matter

VBM analysis on the gray matter and WM images between CW and controls showed no significant differences. See also **Supplementary Tables S1**, **S2** for gray and WM tissue volumes for CW and controls.

## Discussion

CW is a unique case who suffers from unexplained visual field loss with no apparent retinal cause or pre-chiasmal lesions as seen by ERG (ISCEV standard (Marmor et al., 2009), spectral domain OCT, normal function of anterior and posterior visual pathways [visually evoked potentials using the ISCEV standard protocol (Odom et al., 2010)] and a variety of ophthalmological examinations (**Table 1**).

Careful inspection of the structural and functional raw data showed no evidence of structural lesions or atrophy (**Supplementary Figure S1**). However, multiple perimetry tests over the 8 years of our assessments have demonstrated severe anopia of the inferior hemifield, and the upper left visual field, with a clockwise constant progression of the loss through the upper left visual field crossing the vertical meridian, and a moderate anticlockwise progression of the loss in the right hemifield. Remarkably, her visual field loss does not respect the vertical meridian of the visual field corresponding to the lateralization of early retinotopic areas in visual cortex. Typically scotomas respect the vertical meridian because the hemifields are anatomically segregated until relatively high-level areas (Hubel and Wiesel, 1967; Wandell et al., 2007; Martin and Corbett, 2013).

Of great interest is CW’s unusual neuropsychological profile, which illustrates the rarity of the condition. Specifically, while she showed an extreme deficit in a number of simple psychophysical and behavioral paradigms and standardized clinical tests (L-POST), she was able to perform normally in other tests, some of which entailed greater complexity. For example, CW could recognize objects depicted in black and white photographs even when these were presented in unusual views (Warrington and Taylor, 1973); she could not, however, recognize objects in isolation or in a scene (on L POST). Similarly, even though she could not detect shapes or discriminate size in the VOSP and CORVIST tests, she was unimpaired in the shape discrimination and shape ratio discrimination (Efron) tests. It is generally the case that if the shape detection task is impaired, the patient will fail the other tests, and this is stated in the VOSP manual. Still, this discrepancy is another aspect of the unique nature of the visual deficit. It should be noted that similar visual discrepancies can be seen in some disorders especially in the early stages. For example in early stages of posterior cortical atrophy (PCA), patients can identify real objects, while they cannot identify fragmented objects and lines (Maia da Silva et al., 2017). In the early stages PCA patients can fail this test of shape detection task in both the VOSP and CORVIST which involves form detection in spatial noise (a fragmented image) at a time when performance on the Efron test is intact (Maia da Silva, Galton and Plant *in preparation)*. As we have stated this case does not resemble PCA, however, neither in the imaging nor in the visual deficit which has been non-progressive apart from the visual field. In fact, CW’s deficit seems largely to be related to spatial integration: she can recognize a shape defined by individual line segments or Gabor patches easily when they touch but her recognition breaks down when there is any gap. This may be the reason why she can perform relatively complex tasks such as those involving natural images. She is also severely impaired in perceptual grouping of kinetic elements, such as global motion perception and biological motion perception. Furthermore, CW could not perform the motion coherence task, and even when the maximum four out of five dots (or 95 out of 100 dots) moved coherently she could not discriminate the direction of motion. The fact that even subtle amounts of incoherent motion (as well as directional variance) disrupt her ability to discriminate the average direction suggests that her global motion perception is impaired. It should be highlighted that this also is a very unusual finding. For example in a study of 50 stroke patients, 16 of whom had brain lesions including motion area MT/V5, none were unable to perform a motion coherence task (Vaina et al., 2001).

In contrast, CW’s retinotopic mapping data clearly revealed qualitatively normal, well defined retinotopic maps, further supporting the ophthalmological tests performed against a retinal or other pre-chiasmal problem. Specifically, she showed normal retinotopic activation in the dorsal V1–V3 areas corresponding to her most extensive and prolonged visual field loss in the lower right visual field. There were also no obvious differences between intact and blind parts of her visual field in terms of the signal to noise ratio in fMRI responses to the mapping stimulus as estimated by the goodness of fit of the pRF model. While the response amplitude in left dorsal V2 and V3 and right ventral V2 (all corresponding blind parts of her visual field) was weaker than controls, pRF measures exhibit substantial inter-individual differences (Schwarzkopf et al., 2014) and CW’s retinotopic architecture in those regions nonetheless appeared normal. Additional control analyses confirmed that this discrepancy between perimetry and retinotopic mapping results is not trivially explained by an artifact in the pRF modeling procedure.

Additional behavioral measurements of the extent of CW’s visual field using different stimuli (high contrast retinotopic mapping stimulus, Gabor patch orientation discrimination) confirmed the perimetry results and ruled out that the discrepancy between retinotopic mapping and perimetry could have been the result of using different visual stimuli.

Further to her normal functional response in the early visual areas, we found no evidence of any pathological lesion (See **Supplementary Figure S4**) or volume loss in gray and WM tissue (segmented from the T1 scans) compared with our controls (**Supplementary Tables S1**, **S2**). To our knowledge, this is the first study showing progressive visual field loss with no apparent retinal cause nor structural cortical lesion, and normal corresponding cortical activation.

Retinotopically organized scotomas usually follow damage to the striate cortex or the inputs to it (Tong, 2003; Papanikolaou et al., 2014). In most instances, evidence of perimetrically identifiable loss following damage to extrastriate visual cortex is attributed to damage to underlying optic radiations (Stasheff and Barton, 2001; Smirnakis, 2016). The only exception are two cases of homonymous quadrantanopia attributed to damage to V2/3 (Horton and Hoyt, 1991). Of relevance, one study (Slotnick and Moo, 2003) performed fMRI on a patient having an upper homonymous quadrantanopia with a structural lesion. Their results showed normal retinotopic activation in ventral V1 and V2, while there was impaired activation in ventral V3 and V4. They suggested that these results are in agreement with Horton and Hoyt’s model in which they predicted that an isolated extra-striate lesion could cause homonymous quadrantanopia (Horton and Hoyt, 1991). However, CW has no discrete lesion and activation in retinotopic areas appears normal.

Another case, LG, shows fMRI deactivation of visual cortex in response to visual stimulation accompanied by developmental object agnosia, but a normal visual field (Gilaie-Dotan et al., 2009). This gives rise to the question whether a “functional lesion” can cause a visual field loss in addition to the abnormal perception. In LG, deactivation to visual stimulation is found in the intermediate visual cortex (V2–V3), but a robust activation is detected in V1 and more downstream the visual processing hierarchy. The authors attributed this to the presence of different pathways and to different functional selectivity in the extra-striate cortex regions. CW also exhibits a wide range of deficits with presumably intermediary visual processing, such as spatial integration. Her visual field loss notwithstanding, the repertoire of her visual impairments is at least superficially very comparable to LG’s; however, at least the fMRI response to our retinotopic mapping stimulus does not appear abnormal in V2 and V3 but this could also be due to differences in the analysis and stimulation paradigm between these studies.

In primates, the left and right hemifields are anatomically segregated until relatively high-level areas (Hubel and Wiesel, 1967) and post-chiasmal defects respect the vertical meridian. The fact that the progression of CW’s scotoma crossed the vertical meridian unimpeded, may suggest dysfunction in higher visual areas where receptive fields are known to extend well into the ipsilateral visual field (Dumoulin and Wandell, 2008). Unfortunately, our data did not allow detailed investigation of structural analysis such as structural connectivity using diffusion tensor imaging, which could perhaps provide more insight into any underlying cause.

Numerous cases are described and encountered in clinical practice with what is variable termed “non-organic” or “medically unexplained” visual loss (Hurst and Symns, 1919; Griffiths and Eddyshaw, 2004). In those patients debate continues as to the extent to which such visual loss is entirely psychogenic or whether some disturbance of cerebral function may be present. Characteristically, inconsistencies between examinations may be key to the functional/nonorganic nature (Leavitt, 2006) such as variability in performing visual tasks. In CW’s case the consistency of performance across a variety of tests (Humphrey/Goldmann perimetry tests, as well as behavioral controls using ripple stimuli similar to the ones used during retinotopic mapping), the complexity of her deficits, and indeed the gradual progression of her visual field loss over time in such an ordered manner (**Figure 1**) makes this interpretation highly unlikely. It should be noted that the fact that some automated visual field tests (HFA30-2 and 120 point screen) show a relatively high false negative rate indicates a deterioration in performance during the test. This does not invalidate the results provided the overall pattern of the deficit is consistent in serial testing as is largely the case here. In fact, for a patient to show such a consistent progressive defect over such a time scale, with no opportunity to refer back to previous tests on the patient’s part, would be quite exceptional as a non-organic manifestation. However, until an organic cause can be identified this possibility cannot be ruled out entirely.

For example there are a number of characteristics found in non-organic visual field loss, such as crossing of isopters on Goldmann perimetry (Incesu, 2013), star shaped or constricted visual field patterns (Chen et al., 2007; Foroozan, 2012), tunnel vision (Chen et al., 2007), or the visual defect failing to vary according to standard geometry with changes in viewing distance. None of these where present in CW’s case (see Supplementary Visual fields). It should be also highlighted that in CW’s case finding the root of her perceptual deficits is complicated further by her having only one eye, which does not allow comparisons between binocular and monocular visual field testing (e.g., Chen et al., 2007).

Our present results do not show evidence of any functional lesion in CW’s visual cortex; thus, the origin of her progressive visual field loss remains unexplained. It is possible that her deficit arises in higher extrastriate areas which would explain why the progressive vision loss does not respect the vertical meridian. This explanation would also be consistent with her perceptual deficits with spatial integration or global vision. However, we cannot conclusively rule out subtle functional deficits in the early pathway or an inorganic cause. Either way, if CW’s visual field loss is caused by an organic factor, the deficit is subtler that what can be revealed by the techniques we employed. Unfortunately, it is unlikely that further examinations can be carried out on CW, at least using functional neuroimaging, but we hope these findings will prove informative in similar cases identified in the future which might otherwise be deemed psychogenic in nature.

## Author Contributions

CM, MS, GP, and DS conceived and planned the experiments. CM, RS, MJ-G, and LdW performed the experiments. MS and DS created the software for the retinotopic and the pRF analysis. All authors provided critical feedback, helped shape the research, and contributed to the interpretation of the results. CM and DS wrote the manuscript in consultation with MS and GP.

## Conflict of Interest Statement

The authors declare that the research was conducted in the absence of any commercial or financial relationships that could be construed as a potential conflict of interest.

## Footnotes

1 www.gestaltrevision.be/tests

2 http://www.fil.ion.ucl.ac.uk/spm

3 http://surfer.nmr.mgh.harvard.edu/fswiki/

4 http://figshare.com/articles/SamSrf_toolbox_for_pRF_mapping/1344765

5 www.fil.ion.ucl.ac.uk/spm

6 http://www0.cs.ucl.ac.uk/staff/g.ridgway/vbm/get_totals.m

7 http://pcl.missouri.edu/bf-binomial

∗ To better summarize the result of all the tests we have drawn by eye the best fitting straight line to the most superior abnormal point. When both tests (HFA 30-2 and 120 point screen) were available, as seen below, we use the wide field test result (HFA 120). Please refer to the Supplementary Visual Field tests for the results of the individual tests.

## Supplementary Material

FIGURE S1 Example sagittal slices from CW’s high resolution T1 scan. For the figure, we have used the most recent T1 (second MRI session) after skull stripping only. Top rows correspond to the left and bottom rows to the right hemisphere as indicated by the *x* coordinate (*x*: −35.5 to 37.5, *y*: 14.85, *z*: 35) shown underneath the volumes.

FIGURE S2 Response amplitude (β) estimates for the right hemisphere plotted against eccentricity for the ventral and dorsal early visual areas. Red color corresponds to CW, black color, to the three controls. Symbols denote the mean in each eccentricity band. Error bars denote 1 standard error of the mean.

FIGURE S3 Control pRF analysis, maps for polar angle. When the portion of the visual field corresponding CW’s scotoma was masked out, maps in all the visual areas for both CW and a healthy control participant were unsurprisingly severely distorted, possibly with the exception of the ventral left visual cortex corresponding to the part where the stimulus remained visible.

FIGURE S4 CW’s Gray **(A)** and White **(B)** matter images from the segmented T1s at 96 months (1) and 53 months (2).

TABLE S1 Whole brain and occipital lobe grey matter (GM) and white matter (WM) volumes. Mean and standard deviation was calculated based on the tissue volumes of the healthy controls using the Dartel modulated segmented images.

TABLE S2 Whole brain grey matter (GM) and white matter (WM) volumes of the original segmented images after controlling for total intracranial volume (calculated as the sum of GM, WM and Cerebrospinal fluid).

DATA SHEETS S1–S3 Visual fields tests over the 8 years period of testing (2005–2013). The tests are provided in chronological order, and a summary of those is depicted in **Figure 1**. Over the 8 years period CW showed a progression of visual field loss which is largely constant; there is however a degree of inconsistency (possibly some improvement between May/June 2005 and July/December 2008, some spiraling on 16/6/5 and high false negative rate on automated perimetry). The authors consider these to be within the limits of variability of these tests carried out under standard clinical conditions using a variety of testing facilities.
