# Supplementary figures and images for: Unexplained Progressive Visual Field Loss in the Presence of Normal Retinotopic Maps
Source: Front Psychol. 2018 Oct 15;9:1722. doi: 10.3389/fpsyg.2018.01722 (PMC6196317; doi:10.3389/fpsyg.2018.01722)

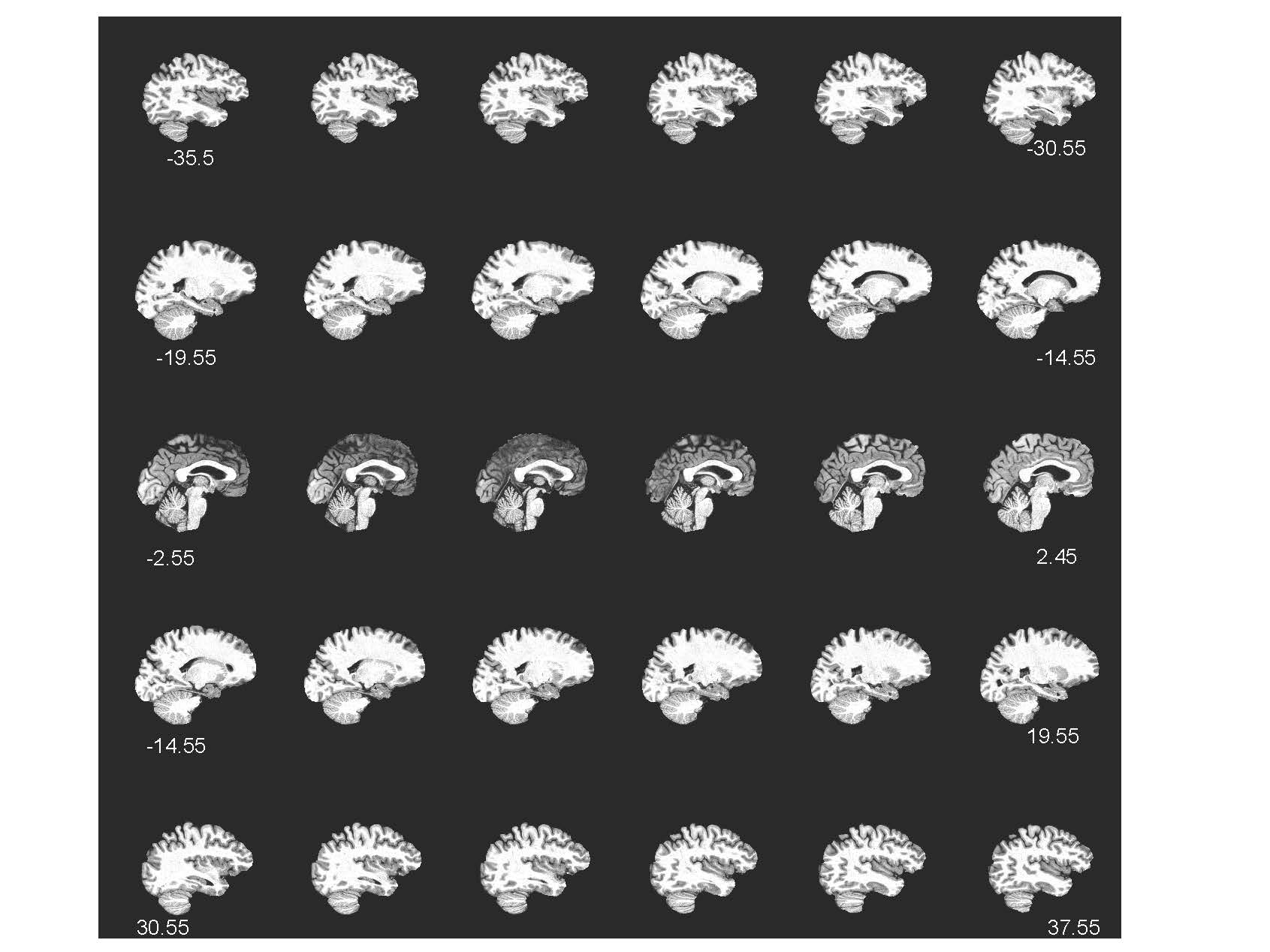

Supplement: FIGURE S1 — Example sagittal slices from CW’s high resolution T1 scan. For the figure, we have used the most recent T1 (second MRI session) after skull stripping only. Top rows correspond to the left and bottom rows to the right hemisphere as indicated by the x coordinate (x: −35.5 to 37.5, y: 14.85, z: 35) shown underneath the volumes. [file Image_1.JPEG]

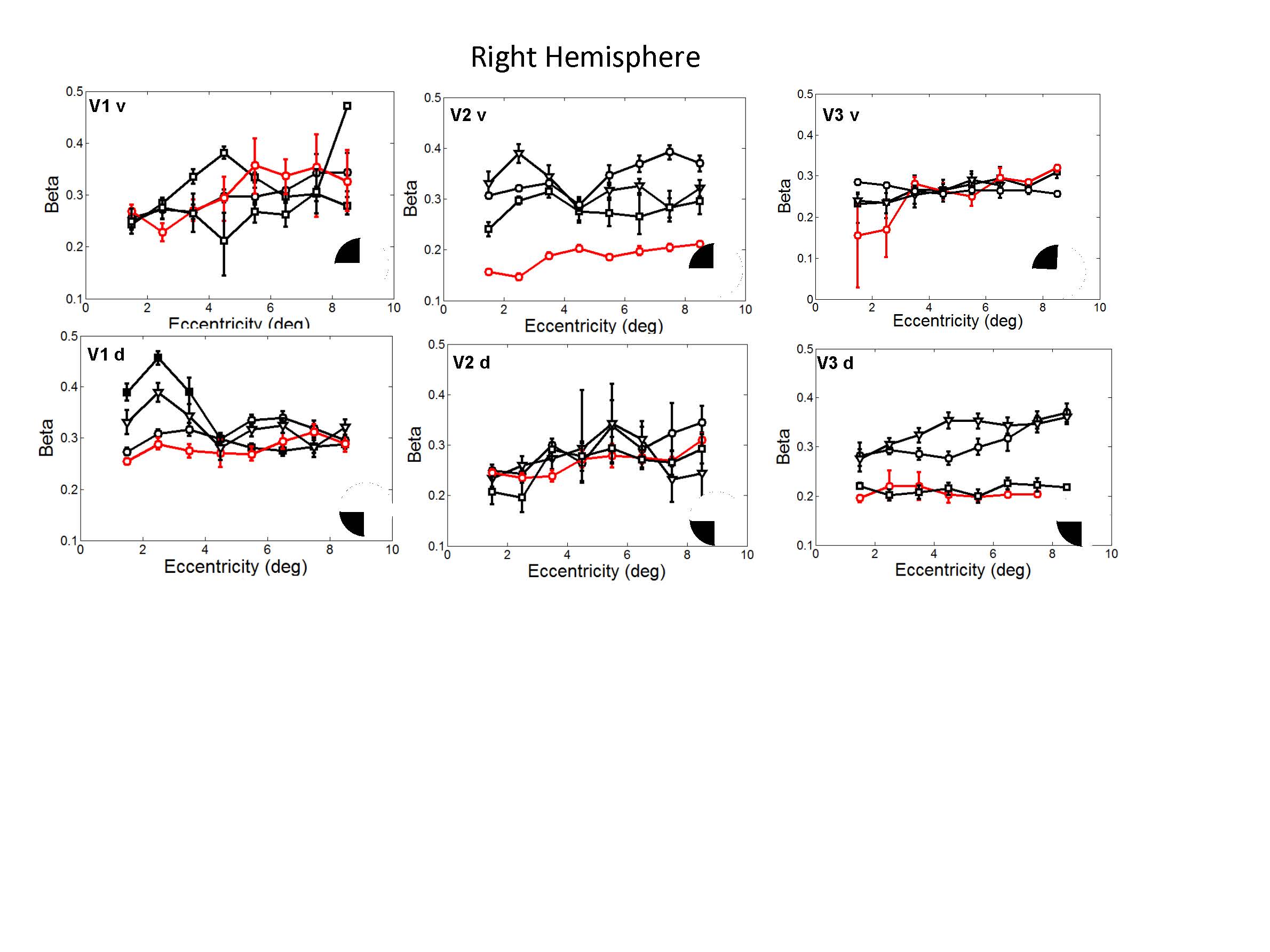

Supplement: FIGURE S2 — Response amplitude (β) estimates for the right hemisphere plotted against eccentricity for the ventral and dorsal early visual areas. Red color corresponds to CW, black color, to the three controls. Symbols denote the mean in each eccentricity band. Error bars denote 1 standard error of the mean. [file Image_2.JPEG]

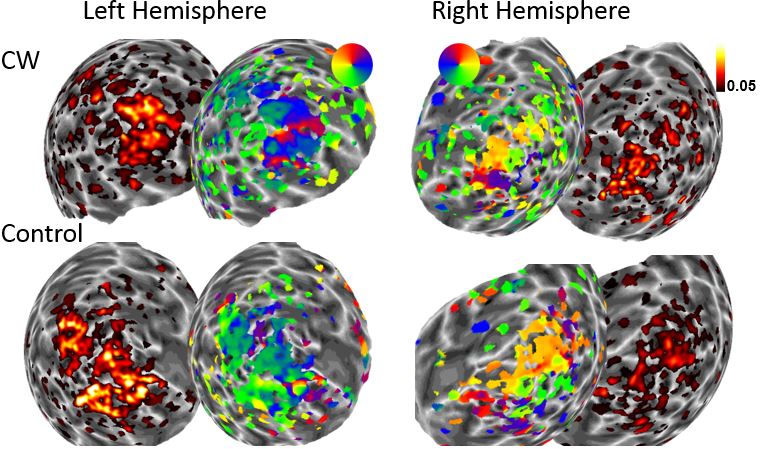

Supplement: FIGURE S3 — Control pRF analysis, maps for polar angle. When the portion of the visual field corresponding CW’s scotoma was masked out, maps in all the visual areas for both CW and a healthy control participant were unsurprisingly severely distorted, possibly with the exception of the ventral left visual cortex corresponding to the part where the stimulus remained visible. [file Image_3.JPEG]

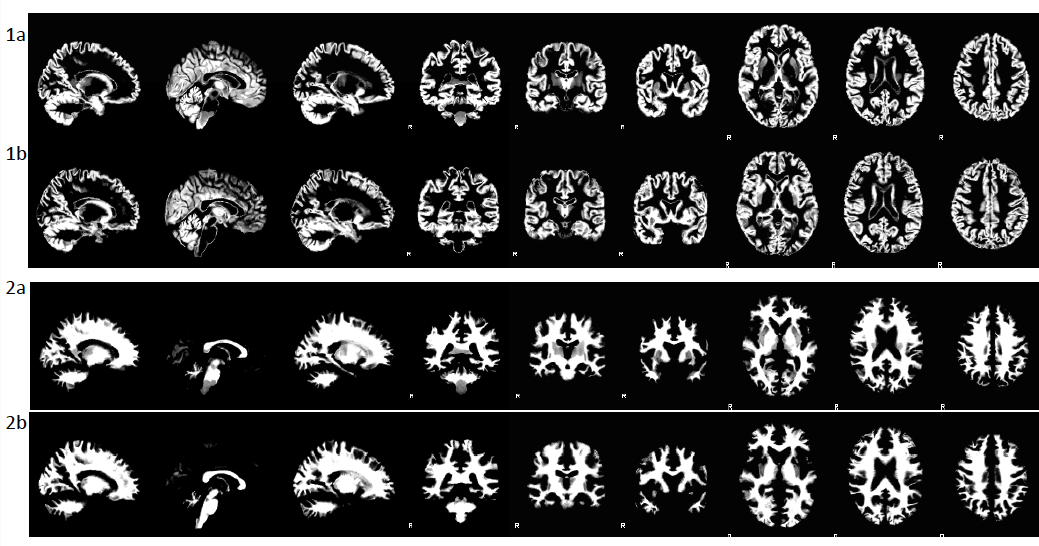

Supplement: FIGURE S4 — CW’s Gray (A) and White (B) matter images from the segmented T1s at 96 months (1) and 53 months (2). [file Image_4.PNG]

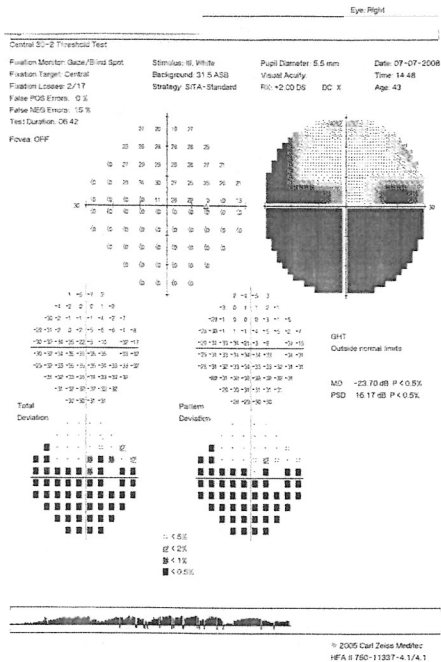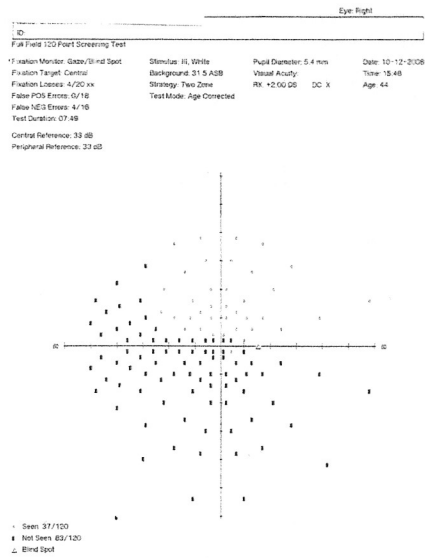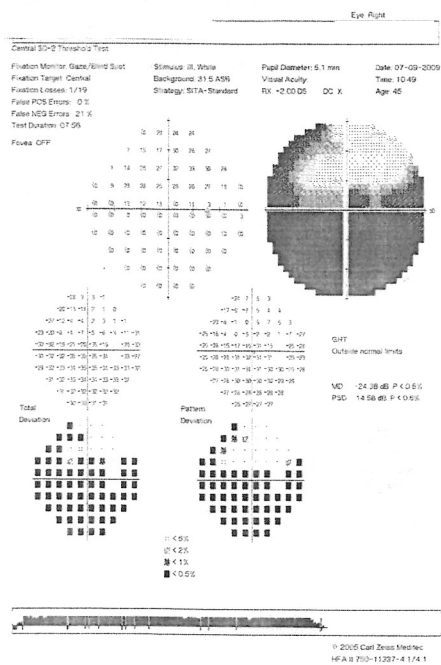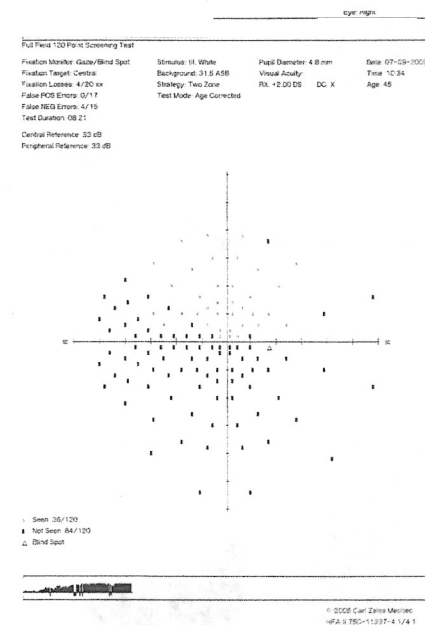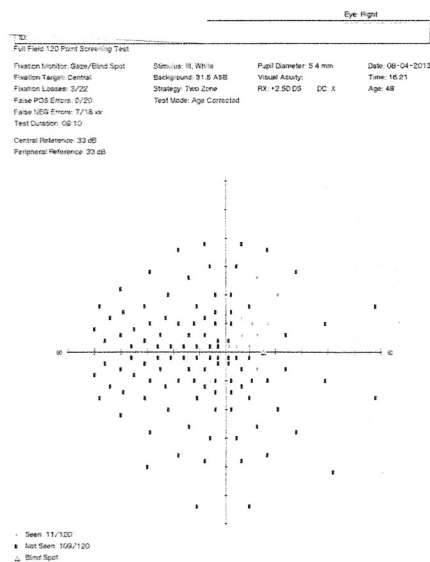

Supplement: DATA SHEETS S1–S3 — Visual fields tests over the 8 years period of testing (2005–2013). The tests are provided in chronological order, and a summary of those is depicted in Figure 1. Over the 8 years period CW showed a progression of visual field loss which is largely constant; there is however a degree of inconsistency (possibly some improvement between May/June 2005 and July/December 2008, some spiraling on 16/6/5 and high false negative rate on automated perimetry). The authors consider these to be within the limits of variability of these tests carried out under standard clinical conditions using a variety of testing facilities. [file Data_Sheet_1.pdf]
