# Supplementary material for: Unexplained Progressive Visual Field Loss in the Presence of Normal Retinotopic Maps
Source: Front Psychol. 2018 Oct 15;9:1722. doi: 10.3389/fpsyg.2018.01722 (PMC6196317; doi:10.3389/fpsyg.2018.01722)
Supplement: TABLE S1 — Whole brain and occipital lobe grey matter (GM) and white matter (WM) volumes. Mean and standard deviation was calculated based on the tissue volumes of the healthy controls using the Dartel modulated segmented images. [file Table_1.pdf]

Suppl.Table 1. Whole brain and occipital lobe grey matter (GM) and white matter (WM) volumes. Mean and standard deviation was calculated based on the tissue volumes of the healthy controls using the Dartel modulated segmented images.

	**GM whole tissue**	**WM whole tissue**	**GM Locc**	**GM Locc**	**GM Rocc**	**GM Rocc**
CW second T1	0.86	0.75	0.54	0.45	0.56	0.44
Mean (st dev) in controls only	0.84 (0.02)	0.70 (0.03)	0.47 (0.06)	0.41 (0.02)	0.493 (0.02)	0.40 (0.01)
CW first T1	*0.78*	*0.62*	*0.48*	*0.40*	*0.50*	*0.40*
