# Supplementary material for: Unexplained Progressive Visual Field Loss in the Presence of Normal Retinotopic Maps
Source: Front Psychol. 2018 Oct 15;9:1722. doi: 10.3389/fpsyg.2018.01722 (PMC6196317; doi:10.3389/fpsyg.2018.01722)
Supplement: TABLE S2 — Whole brain grey matter (GM) and white matter (WM) volumes of the original segmented images after controlling for total intracranial volume (calculated as the sum of GM, WM and Cerebrospinal fluid). [file Table_2.pdf]

Suppl. Table 2. Whole brain grey matter (GM) and white matter (WM) volumes of the original segmented images after controlling for total intracranial volume (calculated as the sum of GM, WM and Cerebrospinal fluid).

	**GM whole tissue**	**WM whole tissue**
CW second T1	0.43	0.30
CW first T1	0.46	0.43
