# Supplementary material for: Unexplained Progressive Visual Field Loss in the Presence of Normal Retinotopic Maps
Source: Front Psychol. 2018 Oct 15;9:1722. doi: 10.3389/fpsyg.2018.01722 (PMC6196317; doi:10.3389/fpsyg.2018.01722)
Supplement: Supplementary file 8 [file Data_Sheet_2.PDF]

## CW performance on the L-POST tests

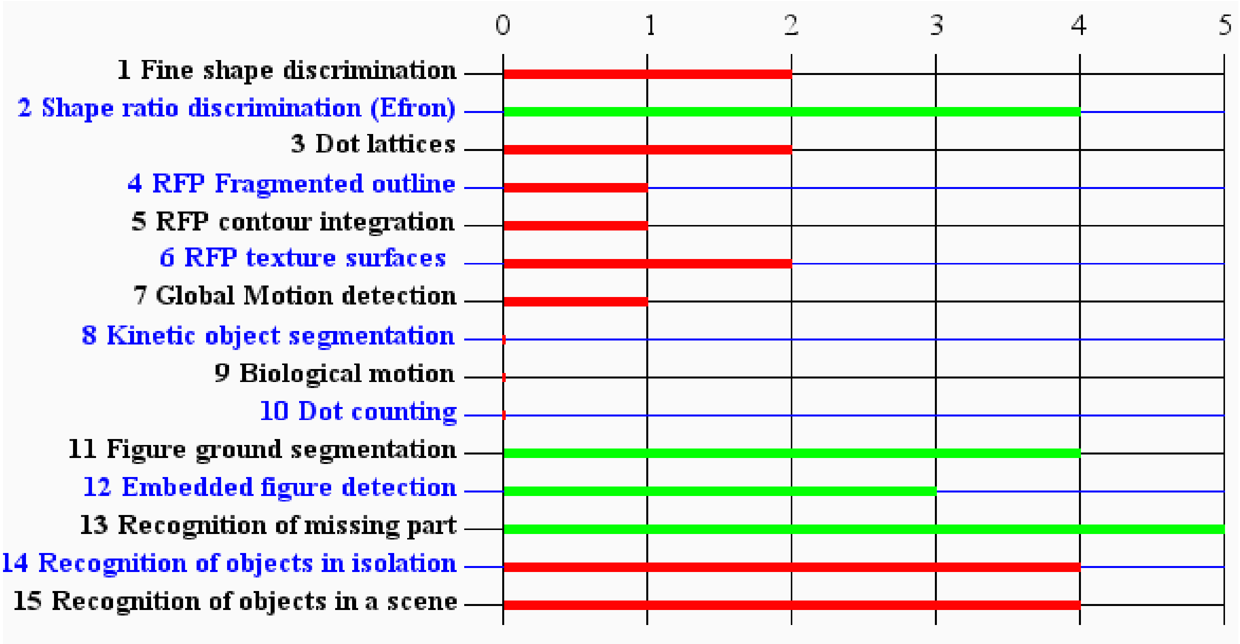


a. CW performance on the L-POST. Test 1

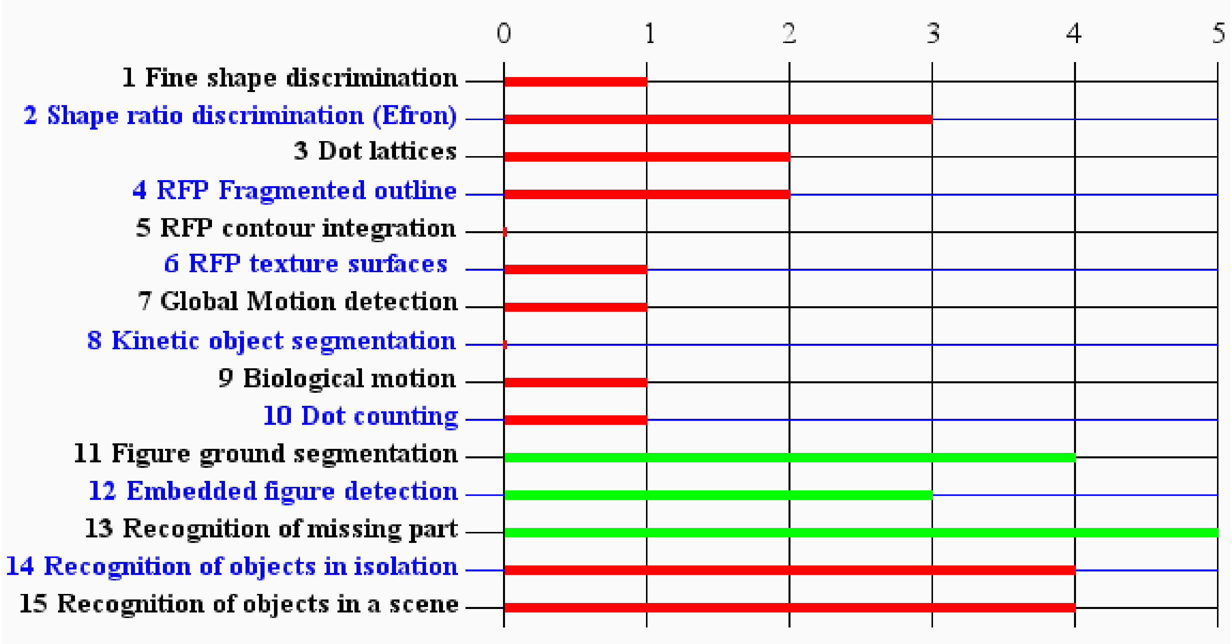


b. CW performance on the L-POST. Test 2

Note: Scores below the 10$^{th}$ percentile are illustrated here in red bar. Performance below the 10$^{th}$ on four or more subtests indicates a deficit. Thus, patient CW scores below the 10th percentile on 12 subtests (a. Test 1). Patients who fail an intermediate number of subtests (4–8) receive further testing (b. Test 2: repetition of the L-POST), to be certain that a deficit has been correctly identified.
