# Supplementary material for: Unexplained Progressive Visual Field Loss in the Presence of Normal Retinotopic Maps
Source: Front Psychol. 2018 Oct 15;9:1722. doi: 10.3389/fpsyg.2018.01722 (PMC6196317; doi:10.3389/fpsyg.2018.01722)

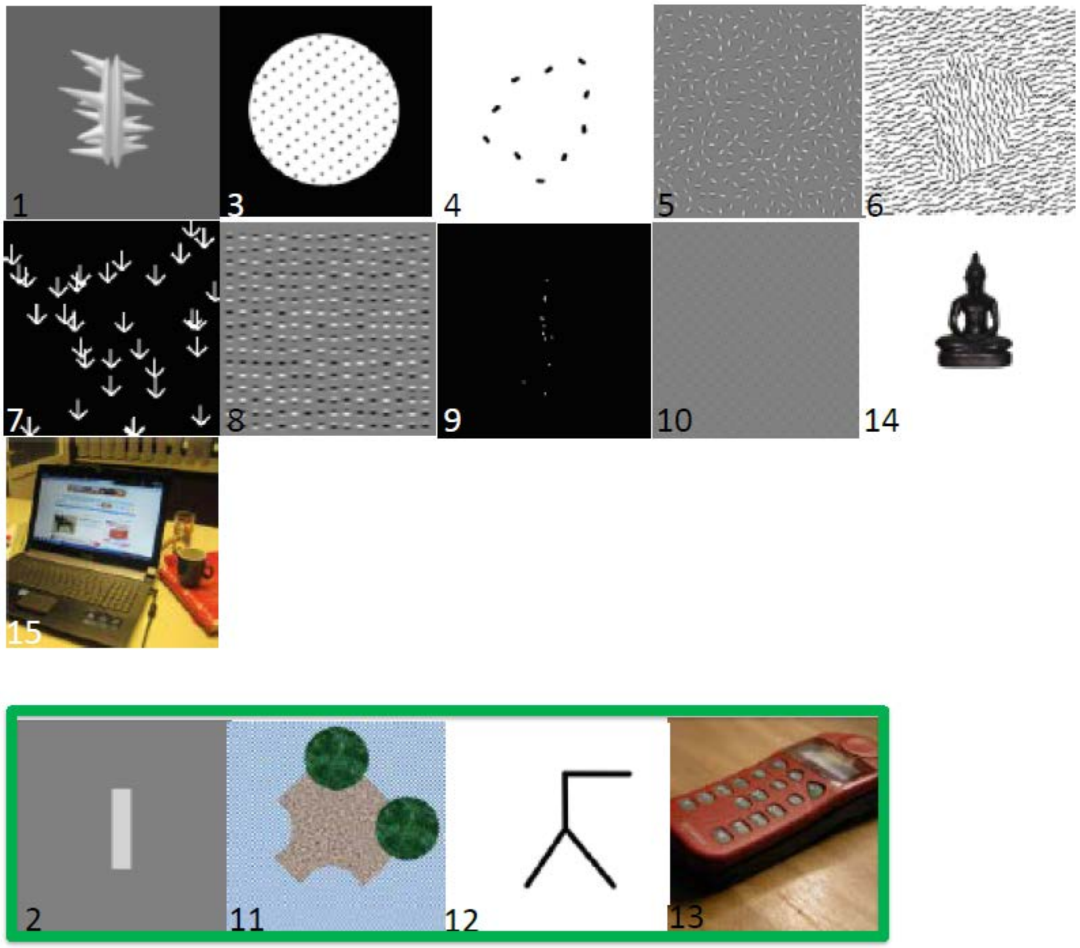

L-POST example stimuli for each of the 15 tasks. CW was only able to perform the ones in the green box.

L POST corresponding tests

1. Fine Shape discrimination — Impaired
2. Shape ratio discrimination (Efron) — Unimpaired
3. Dot Lattices — Impaired
4. RFF fragment Outline — Impaired
5. RFF Contour Integration — Impaired
6. RFF Texture Surfaces — Impaired
7. Global Motion Detection — Extremely Impaired
8. Kinetic Object Segmentation — Extremely Impaired
9. Biological Motion — Extremely Impaired
10. Dot Counting Extremely — Impaired
11. Figure Ground Segmentation using objects — Unimpaired
12. Embedded Figure Detection Segmentation — Unimpaired
13. Recognition Of Missing Part — Unimpaired
14. Recognition Of Object In Isolation — Impaired
15. Recognition Of Object In Scene — Impaired

Visuo-spatial integration example stimuli

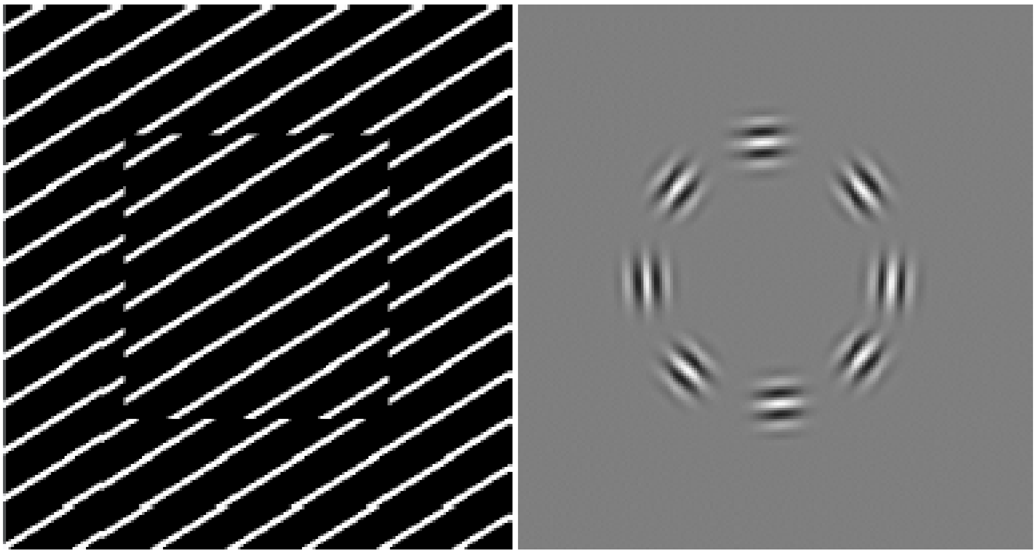

Supplement: Supplementary file 9 [file Data_Sheet_3.PDF]
